# Supplementary material for: Is Colectomy Associated with the Risk of Type 2 Diabetes in Patients without Colorectal Cancer? A Population-Based Cohort Study
Source: J Clin Med. 2021 Nov 15;10(22):5313. doi: 10.3390/jcm10225313 (PMC8622203; doi:10.3390/jcm10225313)
Supplement: Supplementary file 1 [file jcm-10-05313-s001.zip › jcm-1398579-supplementary.pdf]

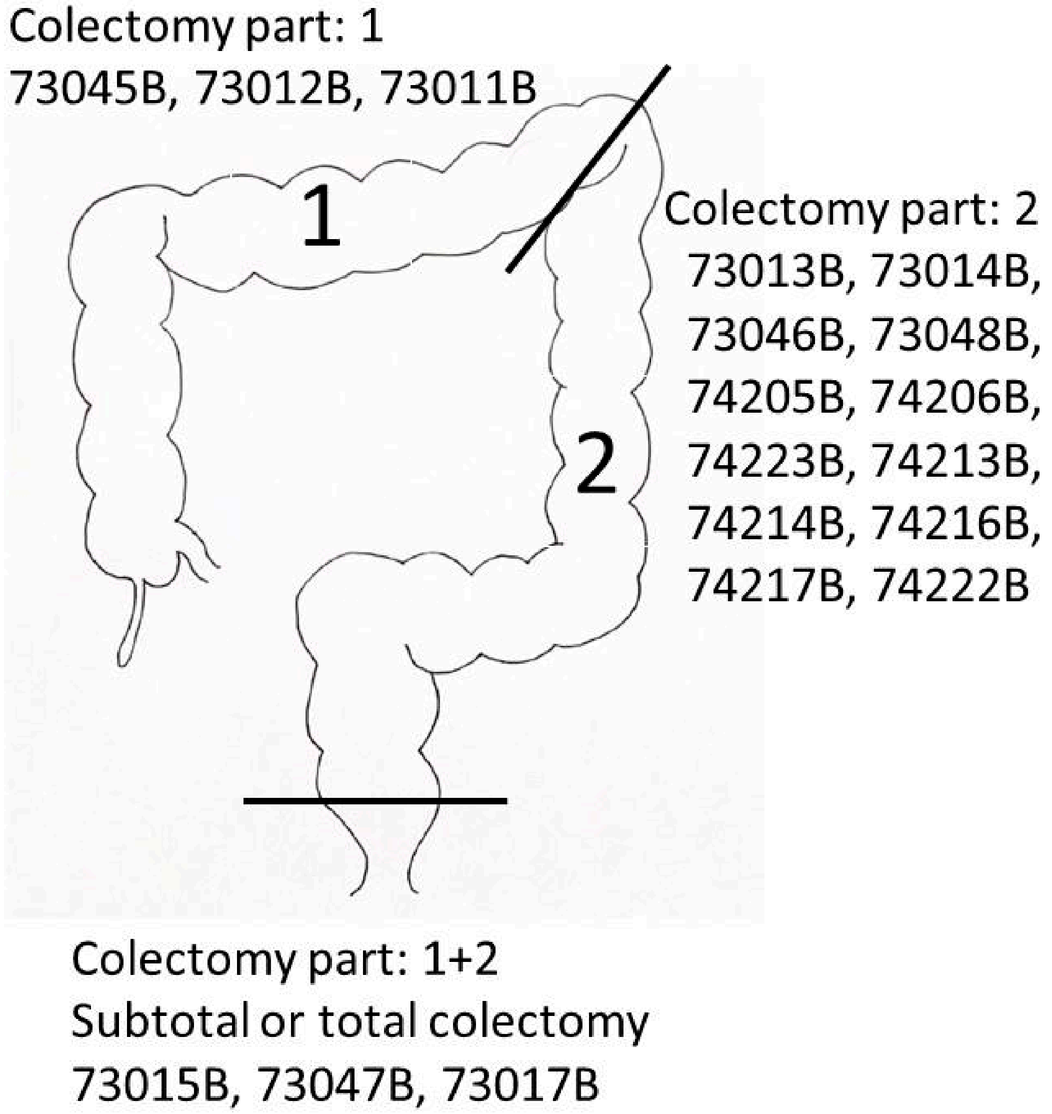

Figure S1. Describe how the procedures categorized by the NHI Treatment Codes.
